# Fruit of *Gardenia jasminoides* Induces Mitochondrial Activation and Non-Shivering Thermogenesis through Regulation of PPARγ

**DOI:** 10.3390/antiox10091418

**Published:** 2021-09-05

**Authors:** Woo Yong Park, Gahee Song, Ja Yeon Park, Kwan-Il Kim, Kwang Seok Ahn, Hyun Jeong Kwak, Jungtae Leem, Jae-Young Um, Jinbong Park

**Affiliations:** 1 Department of Science in Korean Medicine, Graduate School, Kyung Hee University, 26 Kyungheedae-ro, Dongdaemun-gu, Seoul 02447, Korea; parkwy0429@naver.com (W.Y.P.); gahee.ss1@khu.ac.kr (G.S.); wkdusaos5357@naver.com (J.Y.P.); 2 Department of Pharmacology, College of Korean Medicine, Kyung Hee University, 26 Kyungheedae-ro, Dongdaemun-gu, Seoul 02447, Korea; 3 Division of Allergy, Immune Division of Allergy, Immune and Respiratory System, Department of Internal Medicine, College of Korean Medicine, Kyung Hee University, 23 Kyungheedae-ro, Dongdaemun-gu, Seoul 02447, Korea; myhappy78@naver.com; 4 Basic Research Laboratory for Comorbidity Regulation and Department of Comorbidity Research, KyungHee Institute of Convergence Korean Medicine, Kyung Hee University, Seoul 02447, Korea; ksahn@khu.ac.kr; 5 Department of Natural Science, College of Convergence and Integrated Science, Kyonggi University, Suwon 16227, Korea; hjkwak@kyonggi.ac.kr; 6 Research Center of Traditional Korean Medicine, Wonkwang University, 460, Iksan-daero, Sin-dong, Iksan 54538, Jeollabuk-do, Korea

**Keywords:** *Gardenia jasminoides*, non-shivering thermogenesis, beige adipocytes, mitochondria, uncoupling protein 1, peroxisome proliferator-activated receptor gamma

## Abstract

The extract of the *Gardenia jasminoides* fruit (GJFE) can been consumed as an herbal tea or used as a yellow dye. Recently, studies report that GFJE exerts inhibitory effects on lipid accumulation and adipogenesis in white adipocytes. We evaluated the thermogenic actions of GJFE by focusing on mitochondrial activation and studying the underlying mechanisms. To investigate the role of GJFE on thermogenesis in mice, we used an acute cold exposure model. After 2 weeks of feeding, the cold tolerance of GJFE-fed mice was notably increased compared to PBS-fed mice. This was due to an increase in thermogenic proteins in the inguinal white adipose tissue of the cold-exposed mice. Moreover, GJFE significantly increased thermogenic factors such as peroxisome proliferator-activated receptor gamma (PPARγ), uncoupling protein 1 (UCP1), and PPARγ coactivator 1 alpha (PGC1α) in vitro as well. Factors related to mitochondrial abundance and functions were also induced by GJFE in white and beige adipocytes. However, the treatment of PPARγ inhibitor abolished the GJFE-induced changes, indicating that activation of PPARγ is critical for the thermogenic effect of GJFE. In conclusion, GJFE induces thermogenic action by activating mitochondrial function via PPARγ activation. Through these findings, we suggest GJFE as a potential anti-obesity agent with a novel mechanism involving thermogenic action in white adipocytes.

## 1. Introduction

Obesity is a major health threat worldwide and a risk factor of various fatal diseases including type 2 diabetes, cardiovascular diseases, and even cancer [1]. The global incidence of obesity is rapidly increasing [2]. The imbalance between energy intake and expenditure is the main cause of obesity. The contribution of brown adipose tissue (BAT) to energy expenditure has been widely accepted since its unexpected rediscovery in 2007 [3]. White adipose tissue (WAT) stores excess energy as triglycerides; however, BAT induces uncoupled mitochondrial respiration by using fatty acids as fuel to produce heat [4]. This lipid-consumed energy expenditure is considered as a potentially efficient mechanism for obesity treatment [5].

Brown adipocytes increase energy expenditure by activating NTS via uncoupling protein 1 (UCP1). UCP1 converts fatty acids to heat at the mitochondrial inner membrane of brown adipocytes [6]. In addition to conventional brown adipocytes, emerging data have suggested that white adipocytes can also be differentiated into brown-like adipocytes by cold exposure or pharmacological stimulation. These adipocytes are named beige adipocytes, and this endogenous transformation is called ‘browning’ of white adipocytes [7]. Beige adipocytes partially share the characteristics of brown adipocytes such as high UCP1 expression, numerous mitochondria contents and small multilocular lipid droplets and therefore are able to produce heat [8]. The activation strategy of brown/beige adipocytes has been highlighted because of their beneficial action against metabolic diseases including obesity, type 2 diabetes, hypertension, etc. [9]. Moreover, accumulated data suggest a variety of natural products are potential activators of brown and beige adipocyte thermogenesis [10,11].

The fruit of *Gardenia jasminoides* has been long used as a natural source of yellow dye for food and also as medicinal herbs or in the form of tea in Northeast Asia including Korea, China, and Japan [12]. Previous studies have reported that the fruit of *G. jasminoides* (GJF) has various beneficial properties such as anti-inflammation, anti-oxidant, and anti-diabetic effects [12,13,14]. Recent studies also suggest the possibility of GJF as an anti-obesity agent. For instance, Park et al. reported that the ethanol extract of GJF inhibited adipogenesis in white-induced 3T3-L1 cells [15], and Zwirchmayr et al. showed that methylene chloride and methanol extracts of GJF reduced lipid accumulation in *Caenorhabditis elegans* [16]. However, whether GJF has an effect on non-shivering thermogenesis (NTS) is unknown. Here, we investigated the browning effect of GJF and the underlying mechanisms using models of cold-exposed mice, white adipocytes, and beige-induced adipocytes.

## 2. Materials and Methods

### 2.1. Reagents

Dulbecco’s Modified Eagle Medium (DMEM) and fetal bovine serum (FBS) were obtained from Gibco (Waltham, MA, USA). GW9662 and JC-1 were purchased from Cayman Chemical (Ann Arbor, MI, USA). MitoTracker Red was purchased from Thermo Scientific (Waltham, MA, USA). Insulin, 3-Isoutyl-1-methylxanthine (IBMX), dexamethasone, T3, indomethacin, CL316,243, hematoxylin, eosin, and Oil Red O were obtained from Sigma-Aldrich (St. Louis, MO, USA). Cell culture dishes and plates (100 mm dish, 6-well plates, and 96-well plates) were purchased from SPL Life Sciences (Pocheon, Gyeonggi, Korea).

### 2.2. Sample Preparation

Dried GJF was purchased from the Kyung-dong traditional herbal market (Seoul, Korea). The water extract of the GJF (GJFE) was obtained by extracting 50 g of GJF in 1 L of hot water at 100 °C for 3 h, followed by filtering using the Whatman No. 4 (20–25 μm) qualitative filter paper (Kent, UK). After being freeze-dried at −40 °C for 48 h, the powder was dissolved in phosphate-buffered saline (PBS, pH 7.4). Then the GFJE solution was sterilized by a syringe filter (0.2 μm) and used in the experiments. The extraction yield of the GJFE was 11.84% (g/g).

### 2.3. High-Performance Liquid Chromatography (HPLC) Analysis

An Agilent HPLC System 1260 (Agilent Technologies, Inc., Santa Clara, CA, USA) was used to obtain the chromatograms of the GJFE and geniposide. The HPLC analysis was performed with an Agilent Eclipse XDB–C18 column (5 μM pore size and length I.D., 4.6 × 250 mm), water (solvent A), and acetonitrile (solvent B). The chromatograms of the GJFE and geniposide (MW: 388.4 g/mol and PubChem CID: 107848) were observed in the following gradient program: 0–5 min (10% B), 5–20 min (10 to 50% B), 20–35 min (50 to 100% B), 35–40 min (100% B), and 45 min (10% B). Detection was conducted by a diode array detector set at 240 nm. The flow rate was 1 mL/min. The chromatograms of the GJFE and geniposide are shown in Figure S1.

### 2.4. Animal Experiment

All animal procedures were performed according to a protocol approved by the Animal Care and Use Committee of the Institutional Review Board of Kyung Hee University (confirmation number: KHUASP (SE)-19-405). Male C57BL/6J mice (7-week-old, *n* = 20) were purchased from Daehan Biolink Co. (Eumsung, Korea) and kept for 1 week for acclimatization prior to the experiments. The mice were maintained on a 12 h light/12 h dark cycle at 24 °C in a specific pathogen-free environment. The mice (*n* = 20) were randomly divided into two groups: the control group (PBS-fed) and GJFE group (GJFE-fed, 200 mg/kg). All groups of mice were fed on a normal diet and GJFE or PBS was orally administered daily for 2 weeks. After the 14 day administration, 5 randomly selected mice from each group were kept in a cold room maintained at 4 °C for 180 min, and their rectal temperatures were measured using a rectal probe connected to a Testo 925 digital thermometer (Testo Inc., Lenzkirch, Germany) at the indicated time points. After 180 min of cold exposure, the surface temperature of the mice was measured by a thermal imaging camera (FLIR Systems Inc, Wilsonville, OR, USA). Unselected mice (*n* = 5 per group) were kept at RT while the cold exposure experiment was carried out. Then the mice were sacrificed by cervical dislocation under CO_2_ asphyxiation, and the epididymal white adipose tissue (eWAT), inguinal white adipose tissue (iWAT), and BAT were harvested and weighed. The tissues were then either snap frozen and stored at –80 °C for further analysis or fixed in formalin for histological studies.

### 2.5. Cell Culture and Differentiation

The mouse embryo fibroblast cell line 3T3-L1 was obtained from the American Type Culture Collection (Rockville, MD, USA). The cells were maintained in DMEM media supplemented with 10% FBS and 100 U/mL of penicillin and streptomycin in a CO_2_ incubator at 37 °C with 5% CO_2_ until full confluence.

3T3-L1 adipocytes were cultured and differentiated into white adipocytes as described previously [17]. After the cells reached full confluence (day 0), the 3T3-L1 cells were treated with DM (Wh) of DMEM containing 10% FBS and supplemented with 0.5 mM IBMX, 0.5 µM dexamethasone, and 1 µg/mL insulin for two days. The medium was replaced three times with a maintenance medium containing 1 µg/mL insulin every 48 h (at days 2, 4 and 6). After fully differentiated to mature white adipocytes (day 8), the cells were treated with GJFE (125 and 250 µg/mL) or CL316,243 (β3-AR agonist, 1 µM) for 3 days. CL316,243 was used as a positive control for the characteristic transformation to beige-like adipocytes.

3T3-L1 adipocytes were cultured and differentiated into beige adipocytes as previously reported [18]. After 3T3-L1 cells reached full confluence (day 0), the cells were treated with the DM (Be) of DMEM containing 10% FBS and 0.5 mM IBMX, 0.5 µM dexamethasone (Dex), 1 µg/mL insulin, and 50 nM T3 for two days. The medium was replaced three times with a maintenance medium containing 0.5 mM IBMX, 1 µg/mL insulin, 50 nM T3, and 0.5 µM rosiglitazone (Rosi) every 48 h (at days 2, 4 and 6). GJFE was added to the maintenance medium at concentrations of 125 and 250 µg/mL on day 2.

### 2.6. Cell Cytotoxicity Assay

The cytotoxicity was measured with a WST-1 reagent [19]. Briefly, cells were seeded at 3 × 10^4^ cells per well in 96-well plates and maintained for 24 h then incubated with various concentrations of GJFE for 48 h. The absorbance was measured at 490 nm using a VERSAmax microplate reader (Molecular Devices, Sunnyvale, CA, USA).

### 2.7. Oil Red O staining

Intracellular lipid accumulation was measured using Oil Red O [20]. The stain was observed using the EVOSR Cell Imaging System (Thermo Scientific).

### 2.8. Mitochondrial Abundance Assay

Mitochondrial abundance assay was performed as reported previously [21]. Briefly, adipocytes were stained with MitoTracker Red reagent for 30 min. Microscopic examinations were performed under the EVOSR Cell Imaging System. Mitochondrial abundance levels were calculated using the ImageJ software (National Institutes of Health, Bethesda, MD, USA).

### 2.9. Free Fatty Acid (FFA) Assay

Intracellular FFA content was detected using an EZ-free fatty acid assay kit (DoGen Bio, Seoul, Korea) based on the manufacturer’s instructions.

### 2.10. Hematoxylin and Eosin (H&E) Staining

H&E staining was performed as previously reported [22]. Isolated adipose tissues were collected, fixed in 10% paraformaldehyde, and then embedded in paraffin. The sections were stained with hematoxylin and eosin. Microscopic examinations were conducted under the EVOSR Cell Imaging System.

### 2.11. Immunofluorescence Assay

Immunofluorescence assay was performed as previously reported [23]. The cells or tissues were fixed using 10% formalin and blocked with 5% BSA for 1 h and then were incubated with the indicated primary antibody (1:50) overnight at 4 °C. After washing, the cells or tissues were incubated with Alexa Flour 488- or 633-conjugated secondary antibody (1:1000). Fluorescence was detected using the EVOSR Cell Imaging System.

### 2.12. JC-1 Measurement

For immunofluorescent staining of JC-1, the cells were differentiated, stained with 10 µM JC-1 reagent for 30 min at 37 °C, and fluorescence was detected using the EVOSR Cell Imaging System. For flow cytometry analysis of JC-1, the population of red fluorescence (polymer) in GJFE- or PBS-treated beige adipocytes was measured after staining the cells with JC-1 dye (MedChemExpress, LLC., Monmouth Junction, NJ, USA) according to the manufacturer’s instructions.

### 2.13. Western Blot Analysis

Protein extracts were prepared by homogenization in radioimmunoprecipitation assay (RIPA) buffer (Cell Signaling Technology, Danvers, MA, USA). Lysates were resolved by sodium dodecyl sulfate (SDS)-polyacrylamide gel electrophoresis and transferred onto polyvinylidene difluoride (PVDF) membranes (Millipore, Darmstadt, Germany). Then the membranes were blocked and incubated with the indicated primary antibodies (1:1000), followed by incubation with horseradish peroxidase-conjugated secondary antibodies (1:10,000). Protein signals were detected using the ECL advance kit (GE Healthcare Life Sciences, Seoul, Korea).

### 2.14. Statistical Analysis

All data are expressed as the mean ± standard error mean (SEM) of independent experiments. Statistical differences were calculated by unpaired *t*-tests or post-hoc one-tailed Mann–Whitney U tests using Prism 8 (GraphPad Software, San Diego, CA, USA). Probability (*p*) values of < 0.05 were used as the criterion for statistical significance.

## 3. Results

### 3.1. GJFE Induces Thermogenic Action in iWAT of Mice against Acute Cold Exposure

**Figure 1:**
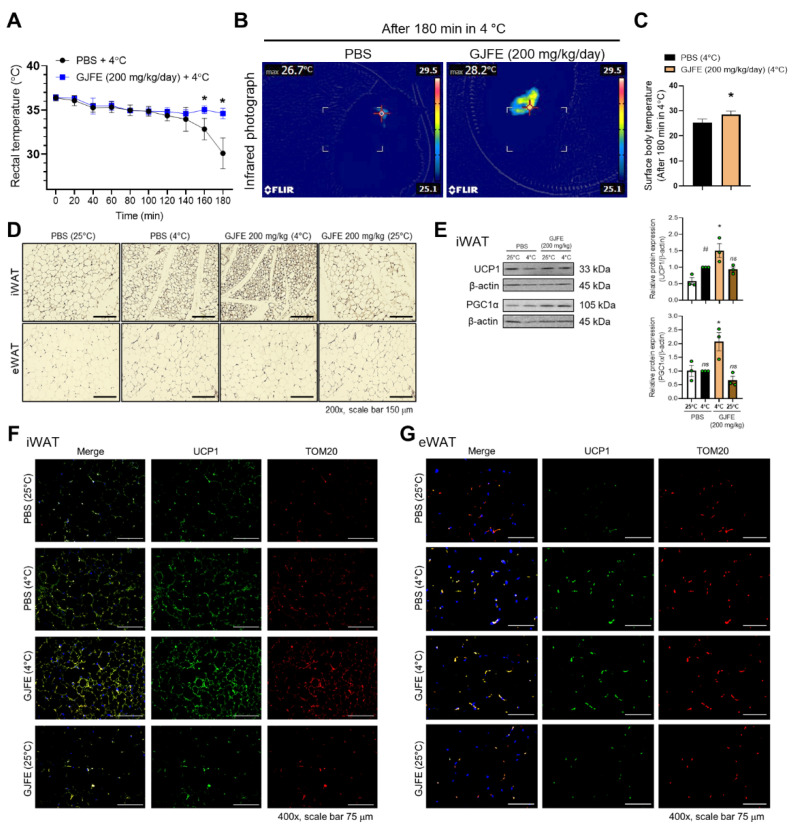
Effect of GJFE on non-shivering thermogenesis in cold-exposed C57BL/J mice. (**A**) Rectal temperature was measured at indicated time points in mice kept in 4 °C or 25 °C for 5 h. (**B**) A representative infrared thermal image and (**C**) average surface temperature are shown (*n* = 5 per group). (**D**) Paraffin-embedded iWAT and eWAT were stained with H&E (magnification 200×, scale bar 150 μm). (**E**) Protein levels of UCP1 and PGC1α were analyzed by Western blot analysis. The expression of UCP1 (green), TOM20 (red), and DAPI (blue) of (**F**) iWAT and (**G**) eWAT in cold-exposed mice were detected by immunofluorescence staining (magnification 400×, scale bar 75 μm). β-actin was used as a loading control for Western blot analysis. ^#^ *p* < 0.05 vs. PBS-fed mice kept in 25 °C, * *p* < 0.05 vs. PBS-fed mice with cold exposure. ns, non-significant. GJFE, *Gardenia jasminoides* fruit extract.

To investigate the effect of the GJFE on NST, we orally administered C57BL/6J mice with GJFE for 12 days and then exposed them to cold at 4 °C for 180 min. After the cold exposure, the rectal temperature of the PBS-fed mice decreased to 30.1 ± 1.6 °C but that of the GJFE-fed mice was relatively stable at 34.6 ± 0.5 °C (Figure 1A). In addition, as shown in Figure 1B,C, the GJFE increased the surface temperature around the BAT region to 28.3 ± 1.2 °C (*p* < 0.05 vs. PBS-fed mice: 25.3 ± 1.2 °C). The GJFE treatment showed a tendency to reduce body weight after 2 weeks (Figure S2A). The cold exposure also seemed to reduce the amount of iWAT, eWAT, and BAT in both the PBS- and GJFE-treated mice (Figure S2B). The adipose tissues (iWAT, eWAT, BAT) were stained with H&E to visualize the morphological change in the lipid droplets of the adipose tissues. The cold exposure led to smaller lipid droplets in the iWAT and GJFE enhanced this change; however, the eWAT was not morphologically affected in all the groups (Figure 1D). The GJFE did not show any significant change in the BAT morphology (Figure S3A). The GJFE treatment increased the protein expression of UCP1 and peroxisome proliferator-activated receptor gamma coactivator 1 alpha (PGC1α) in the iWAT of the cold-exposed mice (Figure 1E). This result was confirmed by immunofluorescence staining. The expression of UCP1 (green) and TOM20 (red) in the iWAT and BAT of the GJFE-fed mice was higher than in the PBS-fed mice (Figure 1F,G, Figure S3B). No change was observed in the eWAT of any of the mice.

### 3.2. GJFE Induces White-to-Beige Trans-Differentiation of White-Induced 3T3-L1 Cells

**Figure 2:**
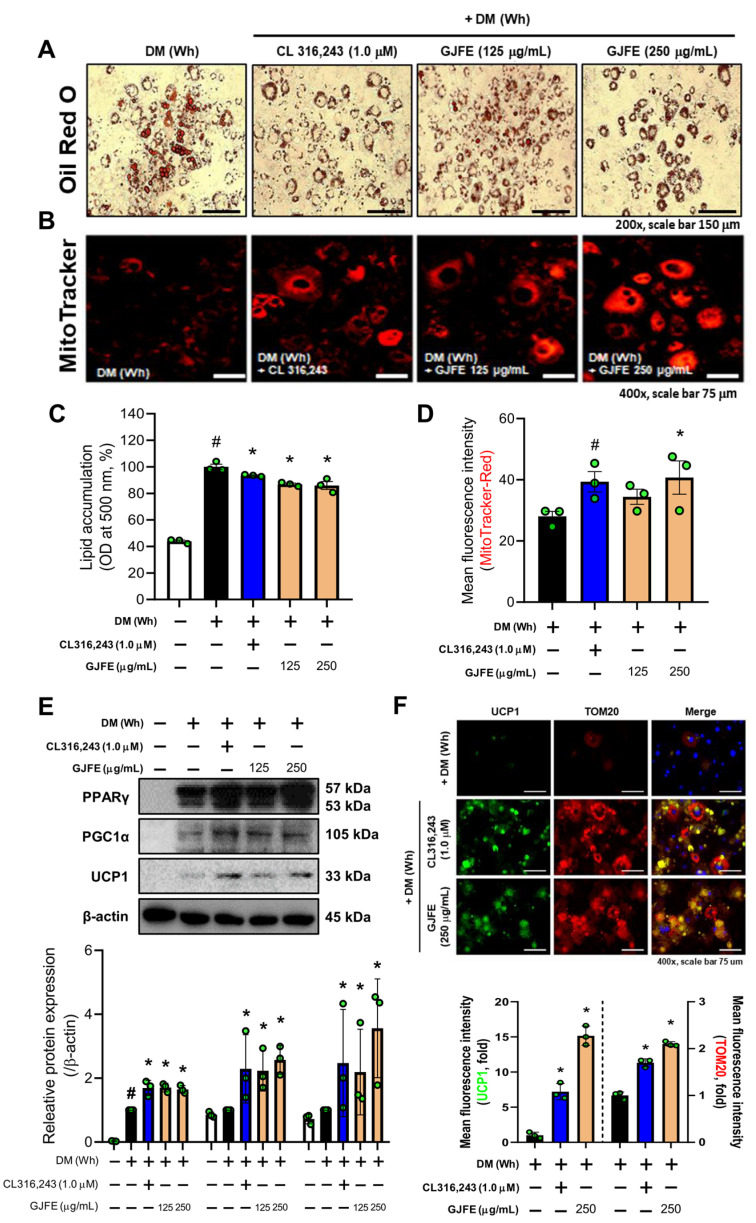
Effect of GJFE on beige trans-differentiation of white-induced 3T3-L1 cells. (**A**) Intracellular lipid droplets were stained with Oil Red O (magnification 400×, scale bar 75 μm), and (**C**) the absorbance was detected at 500 nm in white-induced 3T3-L1 cells treated with GJFE (125 and 250 µg/mL) or CL316,243 (1.0 µM) (β3-AR agonist). (**B**,**D**) Mitochondrial abundance was obtained by MitoTracker Red staining (magnification 400×, scale bar 75 μm). (**E**) Protein levels of PPARγ, PGC1α, and UCP1 were analyzed by Western blot analysis. (**F**) The expression of UCP1 (green), TOM20 (red), and DAPI (blue) were detected by immunofluorescence staining (magnification 400×, scale bar 75 μm). β-actin was used as a loading control for Western blot analysis. All data are expressed as the mean ± SEM of the data from three or more separate experiments. ^#^ *p* < 0.05 vs. DM (Wh)-untreated 3T3-L1 cells, * *p* < 0.05 vs. DM (Wh)-treated 3T3-L1 cells. GJFE, *Gardenia jasminoides* fruit extract.

Next, we used white-induced 3T3-L1 cells to address how GJFE affects the characteristic change of iWAT observed in vivo. The β3-AR agonist CL316,243 was used as a positive control. First, we confirmed no cytotoxicity was observed below 250 µg/mL of GJFE (Figure S4A). The lipid content within the white-induced 3T3-L1 cells was reduced by the treatment of GJFE (Figure 2A,B). GJFE also increased the mitochondrial abundance (Figure 2C,D). Accordingly, we then investigated the thermogenic effect of the GJFE in the white-induced 3T3-L1 cells to see the effect on the beige trans-differentiation of white adipocytes. We confirmed increased protein levels of peroxisome proliferator-activated receptor gamma (PPARγ), PGC1α, and UCP1 in GJFE-treated cells (Figure 2E). We determined that GJFE increases the expression of UCP1 and translocase of outer membrane 20 (TOM20) by immunofluorescence staining as well (Figure 2F). To investigate whether the energy metabolism was increased by GJFE in white adipocytes, we measured the levels of AMP-activated protein kinase (AMPK). GJFE treatment at 250 µg/mL significantly induced the phosphorylation of AMPK to a level similar to that of CL316,243, a β3-AR agonist used as a positive control (Figure S4B). Based on these findings, we could propose that GJFE can induce white-to-beige trans-differentiation of 3T3-L1 cells.

### 3.3. GJFE Activates Thermogenesis in Beige-Induced 3T3-L1 Cells

**Figure 3:**
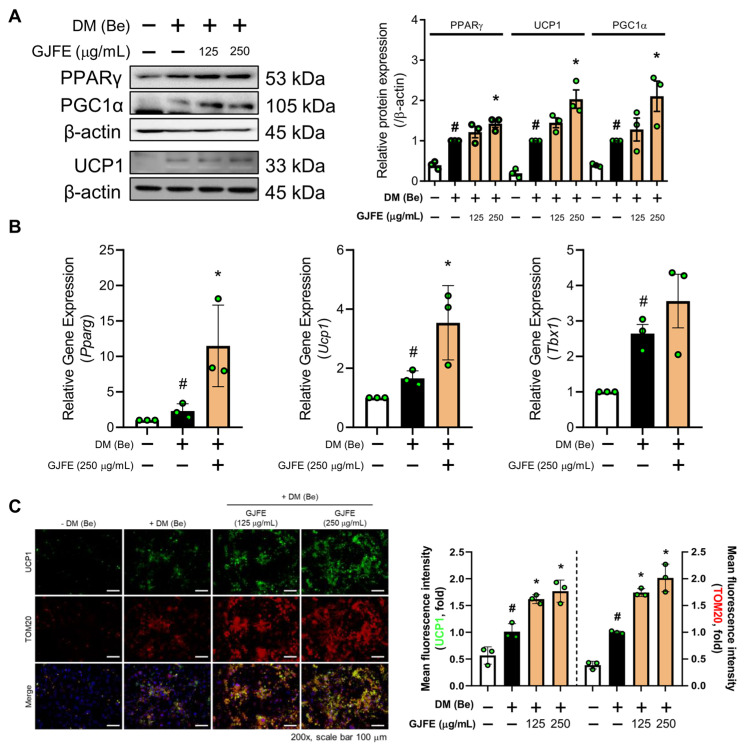
Effect of GJFE on thermogenic markers in beige-induced 3T3-L1 cells. (**A**) Protein levels of PPARγ, PGC1α, and UCP1 were analyzed by Western blot analysis in beige-induced 3T3-L1 cells treated with GJFE (125 and 250 µg/mL). (**B**) mRNA expression of *Ucp1*, *Pparg*, and *Tbx1* in beige adipocytes was measured by RT-PCR analysis and normalized by *Gapdh*. (**C**) The expression of UCP1 (green), TOM20 (red), and DAPI (blue) were detected by immunofluorescence staining (magnification 400×, scale bar 75 μm). β-actin was used as a loading control for Western blot analysis. All data are expressed as the mean ± SEM of the data from three or more separate experiments. ^#^ *p* < 0.05 vs. DM (Be)-untreated 3T3-L1 cells, * *p* < 0.05 vs. DM (Be)-treated 3T3-L1 cells. GJFE, *Gardenia jasminoides* fruit extract.

We studied the potential of GJFE on beige trans-differentiation of white adipocytes above. To see whether GJFE may potentiate the thermogenic activity of beige adipocytes, we next investigated its effect on our previously established beige adipocyte model of 3T3-L1 cells [18]. GFJE significantly increased the protein levels of PPARγ, PGC1α, and UCP1 in the beige-induced 3T3-L1 cells (Figure 3A). Increased mRNA levels of *Ucp1*, *Pparg,* and *Tbx1* were seen as well (Figure 3B). By immunofluorescence staining, we confirmed the increased intracellular expression of UCP1 and TOM20 by GJE in the beige-induced 3T3-L1 cells (Figure 3C). Moreover, AMPK activation was observed (Figure S4C) similarly to the results seen in white adipocytes, indicating upregulation of thermogenesis and related energy metabolism in beige adipocytes as well.

### 3.4. GJFE Induces Lipolysis in Beige-Induced 3T3-L1 Cells

**Figure 4:**
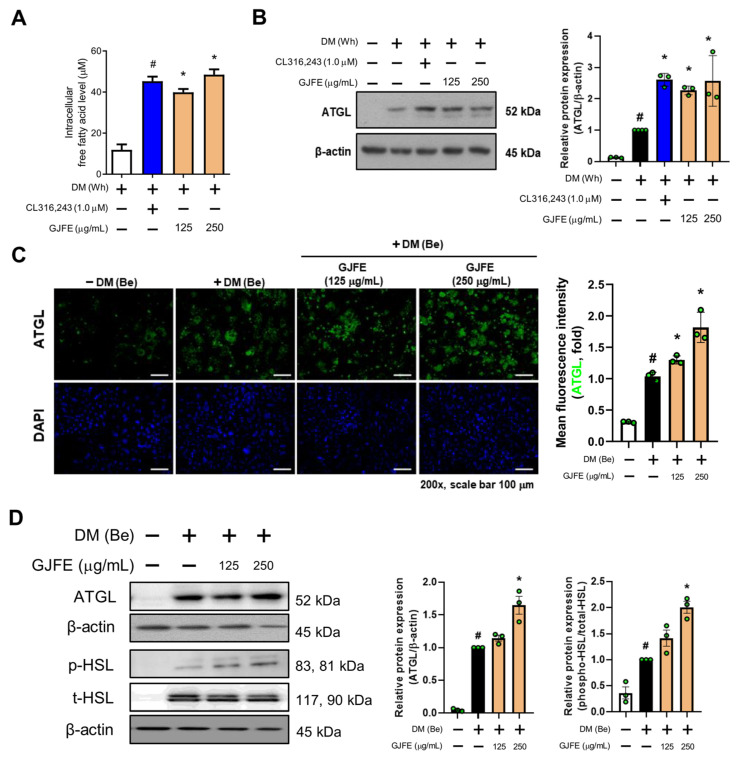
Effect of GJFE on lipolysis markers in differentiated 3T3-L1 cells. (**A**) Level of intracellular free fatty acid and (**B**) protein level of ATGL in white-induced 3T3-L1 cells were analyzed by Western blot analysis. (**C**) ATGL (green) and DAPI (blue) in beige-induced 3T3-L1 cells were detected by immunofluorescence staining (magnification 200×, scale bar 100 μm). (**D**) Protein levels of ATGL, p-HSL, and t-HSL in beige-induced 3T3-L1 cells were analyzed by Western blot analysis. β-actin was used as a loading control for Western blot analysis. ^#^ *p* < 0.05 vs. DM-untreated 3T3-L1 cells, * *p* < 0.05 vs. DM-treated 3T3-L1 cells. GJFE, *Gardenia jasminoides* fruit extract.

Lipolysis is an important process of NST mostly acting as an upstream pathway of it [24]. Increased FFA by GJFE was seen in white adipocytes (Figure 4A); thus, we studied the lipolysis pathway to determine its relevance to the increased FFA. The protein expression of adipose triglyceride lipase (ATGL) was significantly increased upon GJFE treatment (Figure 4B), suggesting a beige trans-differentiation accompanied with elevated lipolysis. We then confirmed the change of lipolysis in the beige-induced cells. GJFE induced an increase in lipolysis markers including ATGL and hormone-sensitive lipase (HSL), which were confirmed by both immunofluorescence staining (Figure 4C) and Western blot analysis (Figure 4D). Through these results, we could confirm that GJFE induces lipolysis in white adipocytes when the thermogenic shift occurs and even potentiates lipolysis in the readily differentiated beige adipocytes.

### 3.5. GJFE Increases Mitochondrial Activation in Beige-Induced 3T3-L1 Cells

**Figure 5:**
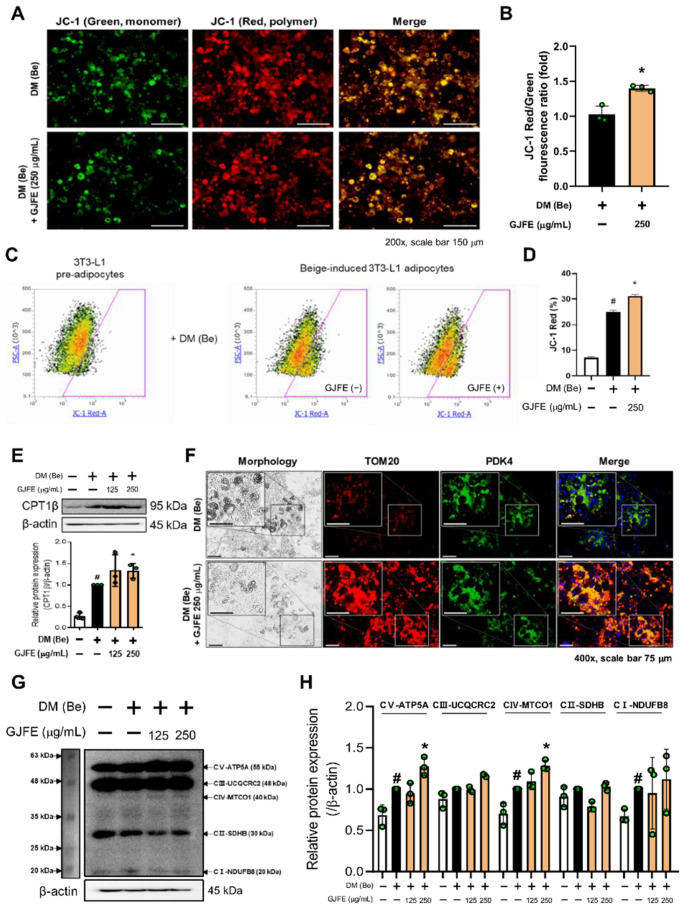
Effect of GJFE on mitochondrial activation in beige-induced 3T3-L1 cells. Mitochondrial membrane potential was measured by JC-1 staining in beige-induced 3T3-L1 cells treated with GJFE (250 µg/mL) by (**A**, **B**) immunofluorescence staining and (**C**, **D**) flow cytometry. (**E**) Protein level of CPT1β was analyzed by Western blot analysis. (**F**) Protein levels of TOM20 (red), PDK4 (green), and DAPI (blue) were detected by immunofluorescence staining (magnification 400×, scale bar 75 μm). (**G**, **H**) Protein levels of OXPHOS complex were analyzed by Western blot analysis. β-actin was used as a loading control for Western blot analysis. All data are expressed as the mean ± SEM of the data from three or more separate experiments. ^#^ *p* < 0.05 vs. DM (Be)-untreated 3T3-L1 cells, * *p* < 0.05 vs. DM (Be)-treated 3T3-L1 cells. GJFE, *Gardenia jasminoides* fruit extract.

Because the abundance and activity of mitochondria in beige/brown adipocytes are directly proportional to the capacity of thermogenesis [25], we investigated whether GJFE regulates mitochondrial activity. JC-1 staining revealed that GJFE increased mitochondrial membrane potential in the beige-induced 3T3-L1 cells (Figure 5A,B). This was further confirmed by flow cytometry (Figure 5C,D). Shown in Figure 5E, the GJFE treatment also enhanced the protein expression of carnitine palmitoyltransferase I beta (CPT1β). Next we investigated the mitochondrial pyruvate dehydrogenase kinase isoenzyme 4 (PDK4) and the OXPOHS complex in the beige-induced 3T3-L1 cells. The expression of mitochondrial PDK4 (merged color yellow) was increased by GJFE (Figure 5F). In addition, the GJFE significantly increased the protein levels of MTCO1 (complex 4) and ATP5A (complex 5) as well (Figure 5G,H).

### 3.6. GJFE Suppresses Reactive Oxygen Species (ROS) in Beige-Induced 3T3-L1 Cells

**Figure 6:**
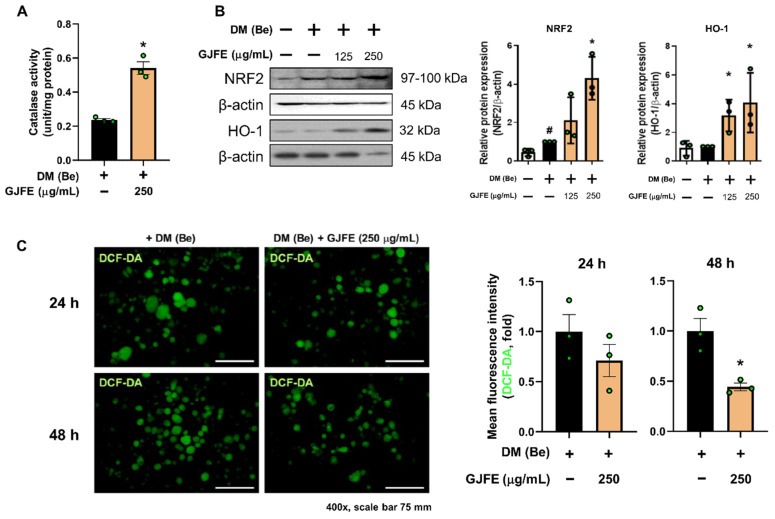
Effect of GJFE on oxidative stress in beige-induced 3T3-L1 cells. (**A**) Catalase activity was measured in beige-induced 3T3-L1 cells treated with GJFE (250 µg/mL). (**B**) Protein levels of NRF2 and HO-1 were analyzed by Western blot analysis. (**C**) DCF-DA staining was performed at 24 h and 48 h post GJFE treatment in beige-induced 3T3-L1 cells (magnification 400×, scale bar 75 μm). β-actin was used as a loading control for Western blot analysis. All data are expressed as the mean ± SEM of the data from three or more separate experiments. ^#^ *p* < 0.05 vs. DM (Be)-untreated 3T3-L1 cells, * *p* < 0.05 vs. DM (Be)-treated 3T3-L1 cells. GJFE, *Gardenia jasminoides* fruit extract.

ROS have an important role in cellular physiology. Oxidative stress negatively impacts metabolism by affecting enzyme activities [26]. Thus WAT ROS levels are typically increased in obese individuals [27]. We therefore investigated the change in ROS and relevant enzymes. As shown in Figure 6A, we found that GJFE significantly increased the activity of the antioxidant enzyme catalase. In addition, markers that are known to encounter oxidative stress such as NRF2 and HO-1 were suppressed by GJFE (Figure 6B). Intracellular ROS was examined by DCF-DA staining. As in Figure 6C, GJFE markedly decreased the intracellular ROS levels, especially showing statistical significance after 48 h of treatment (*p* < 0.05).

### 3.7. Thermogenic Effect of GJFE in Beige Adipocytes Depends on the PPARγ Activation

**Figure 7:**
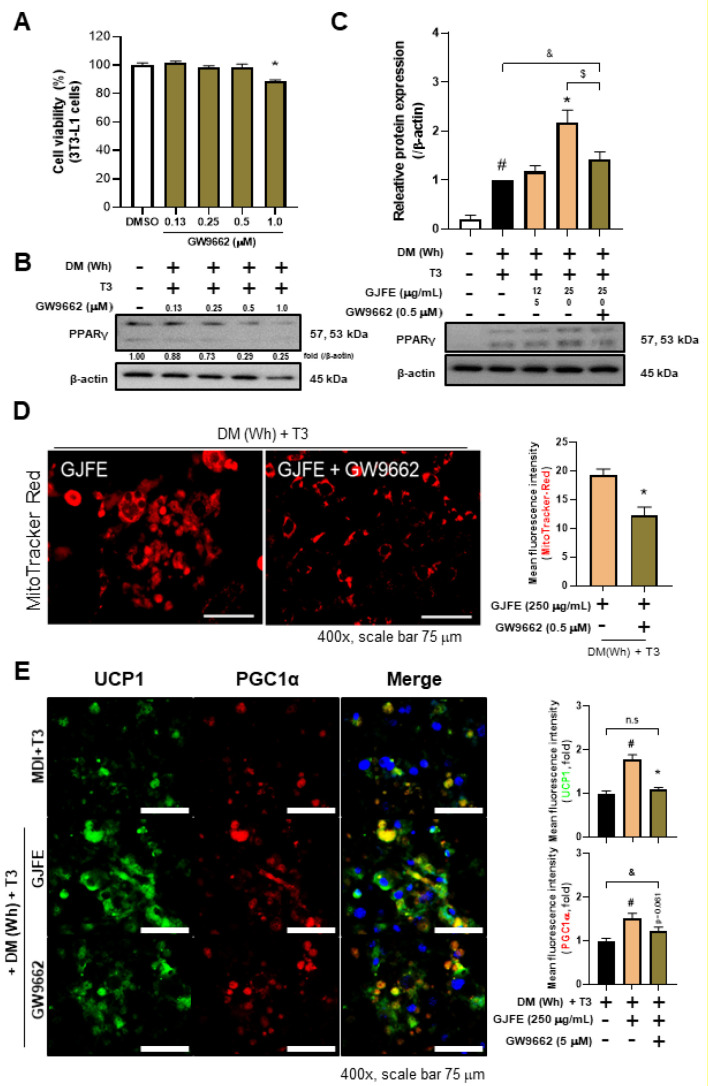
Role of PPARγ in GJFE-induced thermogenic action of 3T3-L1 adipocytes. (**A**) Cytotoxicity of GW9662 (0.13 to 1.0 µM) was measured in 3T3-L1 cells. (**B**) Protein expression of PPARγ in GW9662-treated cells was analyzed by Western blot analysis. (**C**) PPARγ levels of GJFE (125 and 250 µg/mL)-treated 3T3-L1 cells in the presence/absence of GW9662 (0.5 µM) were analyzed by Western blot analysis. (**D**) Mitochondrial abundance was detected by MitoTracker Red staining (magnifica-tion 400×, scale bar 75 µm). (**E**) Protein expressions of UCP1 (green) and PGC1α (red) were detected by immunofluorescence staining (magnification 400×, scale bar 75 µm). β-actin was used as a loading control for Western blot analysis. All data are expressed as the mean ± SEM of the data from three or more separate experiments. ^#^ *p* < 0.05 vs. DM (Wh) + T3-untreated 3T3-L1 cells, * *p* < 0.05 vs. MDI + T3-treated 3T3-L1 cells, ^$^ *p* < 0.05 vs. DM (Wh) + T3 + GJFE, ^&^ *p* < 0.05 vs. DM (Wh) + T3 + GJFE + GW9662. GJFE, *Gardenia jasminoides* fruit extract. DM (Wh), IBMX + Dex + insulin. T3, triiodothy-ronine.

We found that GJFE markedly increased PPARγ in the beige- and white-induced 3T3-L1 cells, thus we sought to investigate its detailed molecular mechanism. First, to confirm the significance of PPARγ on UCP1 expression, 3T3-L1 cells were differentiated in the presence/absence of the PPARγ agonist Rosi. The absence of Rosi during differentiation resulted in a markedly reduced expression of both the *Pparg* and *Ucp1* genes (Figure S5), suggesting that PPARγ is an important factor of thermogenic alteration in adipocytes. The PPARγ inhibitor GW9662 did not affect the viability of the 3T3-L1 cells at concentrations lower than 0.5 µM (Figure 7A). GW9662 indeed suppressed the PPARγ expression in the beige-induced 3T3-L1 adipocytes in a concentration-dependent manner (Figure 7B). To confirm whether the thermogenic effect of GJFE is PPARγ-dependent, we treated the beige-induced 3T3-L1 cells with GJFE after pre-treatment with GW9662. The increase in PPARγ by GJFE was revoked by GW9662 (Figure 7C). In addition, the GJFE-induced increase in mitochondrial abundance was rather suppressed in the presence of GW9662 (Figure 7D). GW9662 also abrogated the enhancing effect of GJFE on the thermogenic proteins (UCP1 and PGC1α) (Figure 7E).

## 4. Discussion

Beige adipocytes are derived from two different processes: from white-to-beige trans-differentiation and from de novo beige differentiation from adipocyte precursors [28]. Recent studies have suggested that natural products and their compounds possess the potential to induce UCP1-dependent thermogenesis by recruiting beige adipocytes in iWAT [17,18,22]. Consistent with this, this study showed the properties of GJFE on inducing beige trans-differentiation of white adipocytes and activating thermogenesis. We studied the underlying mechanisms of GJFE using cold-exposed mice, white-to-beige trans-differentiation in mature adipocytes, and beige differentiation from preadipocytes.

The capacity of energy expenditure of brown and beige adipocytes is decided by mitochondrial quality and subsequent UCP1 expression [29]. During the process of beige differentiation, the coordination of PPARγ, PR domain containing 16 (PRDM16), and PGC1α regulates the transcriptional control of thermogenic genes to increase the accumulation of UCP1 and other beige-related proteins. Meanwhile, to maintain the thermogenic program in brown and beige adipocytes, an abundant supply of the FFAs is also fundamental [30]. FFAs are produced mainly through ATGL-mediated hydrolysis of intracellular lipid droplets. GJFE treatment increased the lipolysis markers ATGL and HSL [31]. Mechanistically, FFAs in the cytosol transfer into the inner membrane by the action of membrane transporters such as CPT1 [32], and sequentially undergo oxidation for the production of ATP or heat [33]. The abundance of mitochondrial FFAs is regulated by PDK4 activation and is negatively associated with the glycolysis pathway [34]. In short, the thermogenic action of brown and beige adipocytes is accelerated by a complex process involving both FFA metabolism and UCP1 expression. Our results showed that GJFE increases thermogenic proteins such as UCP1 and PGC1α in cold-exposed iWAT and 3T3-L1 adipocytes. In addition, the expression of mitochondrial proteins including TOM20, CPT1β, and PDK4 was remarkably increased by GJFE. Other markers, such as JC-1, also supported the mitochondrial activation by the GJFE treatment. AMPK is a crucial regulator of energy metabolism [35] and also a mediator of the beige induction of white adipocytes [36]. GJFE induced the activation of AMPK as well. Thus, the effect of GJFE on mitochondria-mediated NST activation was confirmed in vivo and in vitro.

PPARs are members of the nuclear receptor family which act as master regulators of adipocyte differentiation and lipid metabolism in adipocytes [37]. PPARγ has a key role in de novo lipogenesis by orchestrating the expression of CCAAT enhancer-binding protein alpha (CEBPα) in WAT [38]. PPARα is known to induce lipid metabolic pathways including lipolysis and thermogenesis through ATGL and CPT1 in brown adipose tissue (BAT) [39]. PPARγ is also one of the main regulators in beige/brown adipocyte-mediated thermogenesis [40]. Recent studies suggest that selective PPARγ activating thiazolidinediones (TZDs) such as Rosi can induce BAT activation and WAT browning [40,41]. Results from the in vitro and in vivo models showed this happens through the PPARγ-mediated regulation of PRDM16 and PGC-1α [42,43]. PPARs indeed have an important role in the action of GJFE. In particular, we found that PPARγ is required for the activation of thermogenesis by the GJFE treatment.

Obesity is a metabolic disease typically accompanying excessively increased ROS levels and mitochondrial dysfunction of adipocytes, which result in the pathological hypertrophy of white adipocytes [44]. On the other hand, brown and beige adipocytes, which can improve obesity, have been shown to be rich in mitochondria and have relatively higher mitochondrial ROS levels compared to white adipocytes [45]. Importantly, emerging data show that mitochondrial ROS in brown adipocytes is required for differentiation and activation [46]. When thermogenesis occurs in brown and beige adipocytes, NRF2 has been suggested as an important mediator of redox homeostasis, which induces the accumulation of antioxidant enzymes including catalase, SOD, HO-1, and NQO1 [47,48]. Our results showed that the ROS levels were increased upon beige differentiation consistent with previous reports. Unexpectedly, the GJFE treatment significantly decreased these ROS increases and induced NRF2 and HO-1. Yet no negative effect on thermogenesis accompanied the decrease in ROS. Considering the complicated role of ROS and the related pathways in brown/beige adipocyte thermogenesis, these results together may suggest the decrease in ROS is a defensive effect of the GJFE against oxidative damage in adipocytes.

## 5. Conclusions

Taken together, GJFE induces thermogenic action by activating mitochondrial function. Importantly, the PPARγ level is significantly increased by GJFE, and through investigation regarding Rosi and GW9662, we confirmed that the thermogenic effect of GJFE is dependent on PPARγ activation. Further study is necessary to clarify the full action mechanism of GJFE, and substantial work is necessary to determine the respon-sible compound that exerts such a thermogenic effect. However, the findings in this study advance our understanding on the pharmacological role of GJFE in thermogenic action. Such evidence may enable GJFE to be used as a PPARγ-dependent thermogenic agent, which targets adipocyte browning and therefore decreases lipid accumulation in adipocytes.

## Data Availability

The data presented in this study are available on request from the corresponding author. The data are not publicly available due to currently ongoing patent application.

## Supplementary Materials

The following are available online at https://www.mdpi.com/article/10.3390/antiox10091418/s1. Figure S1: HPLC analysis of geniposide in GJFE. Figure S2: Effect of GJFE on body and tissue weight in cold-exposed mice. Figure S3: Effect of GJFE on BAT in cold-exposed mice. Figure S4: Effect of GJFE on cytotoxicity and protein levels of AMPK in 3T3-L1 adipocytes. Figure S5: Effect of PPARγ agonist on mRNA expression of UCP1 in beige adipocytes.
