# Supplementary material for: Fruit of Gardenia jasminoides Induces Mitochondrial Activation and Non-Shivering Thermogenesis through Regulation of PPARγ
Source: Antioxidants (Basel). 2021 Sep 5;10(9):1418. doi: 10.3390/antiox10091418 (PMC8466082; doi:10.3390/antiox10091418)
Supplement: Supplementary file 1 [file antioxidants-10-01418-s001.zip › antioxidants-1338908-supplementary.pdf]

Supplemental data

Fruit of *Gardenia jasminoides* induces mitochondrial activation and non-shivering thermogenesis through regulation of PPARγ

Park et al.

Figure S1

Figure S2

Figure S3

Figure S4

Figure S5

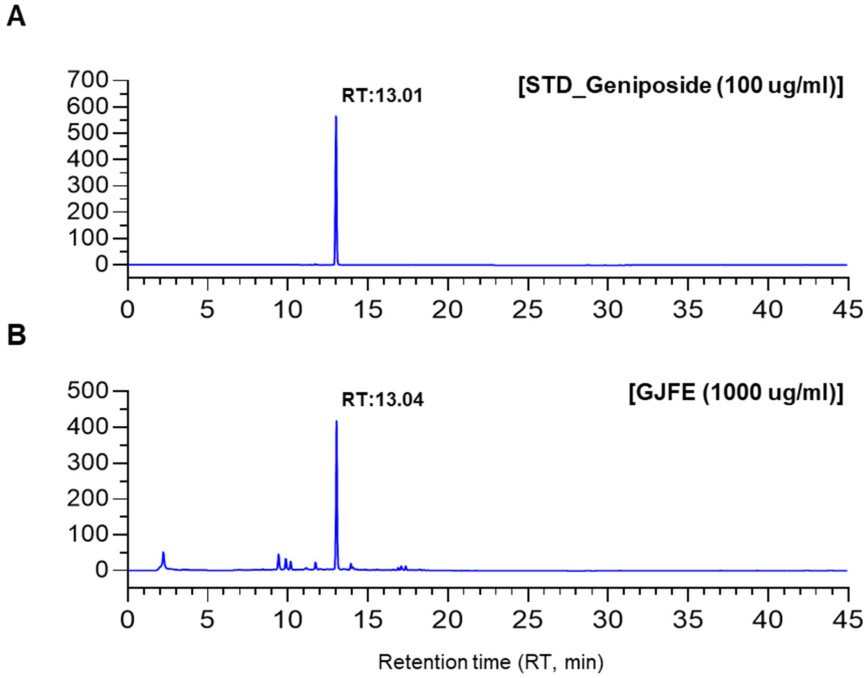

Figure S1. HPLC analysis of geniposide in GJFE. Chromatograms of (**A**) geniposide and (**B**) GJFE were exhibited by HPLC analysis. GJFE, *Gardenia jasminoides* fruit extract.

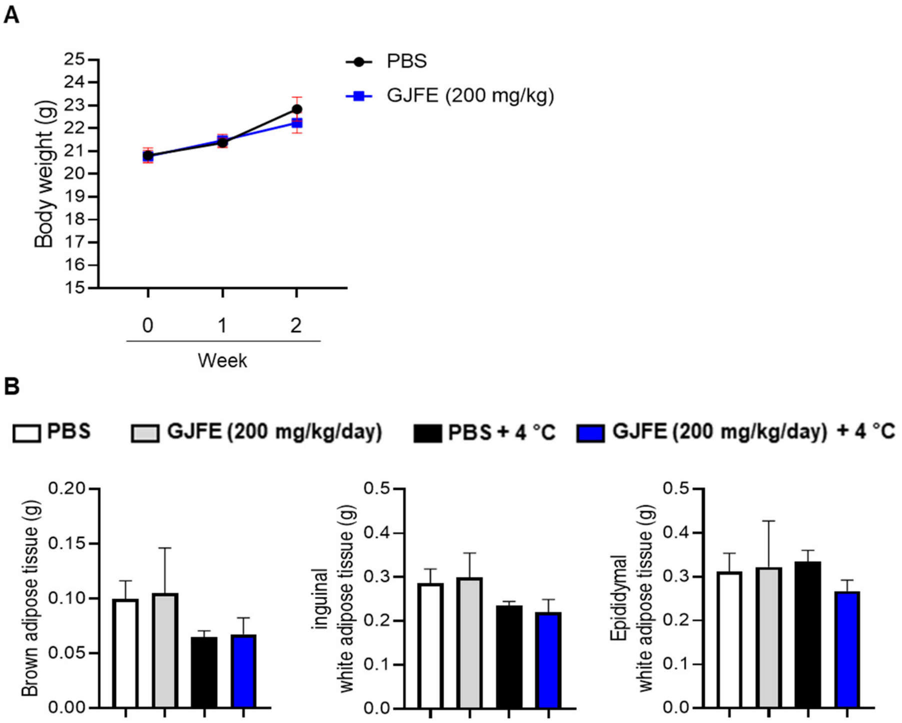

Figure S2. Effect of GJFE on body and tissue weight in cold-exposed mice. (**A**) Body weight of PBS-fed or GJFE-fed mice (*n* = 10) was measured at indicated time points. (**B**) Weight of BAT, iWAT, and eWAT was measured (*n* = 5). GJFE, *Gardenia jasminoides* fruit extract; BAT, brown adipose tissue; iWAT, inguinal white adipose tissue; eWAT, epididymal white adipose tissue.

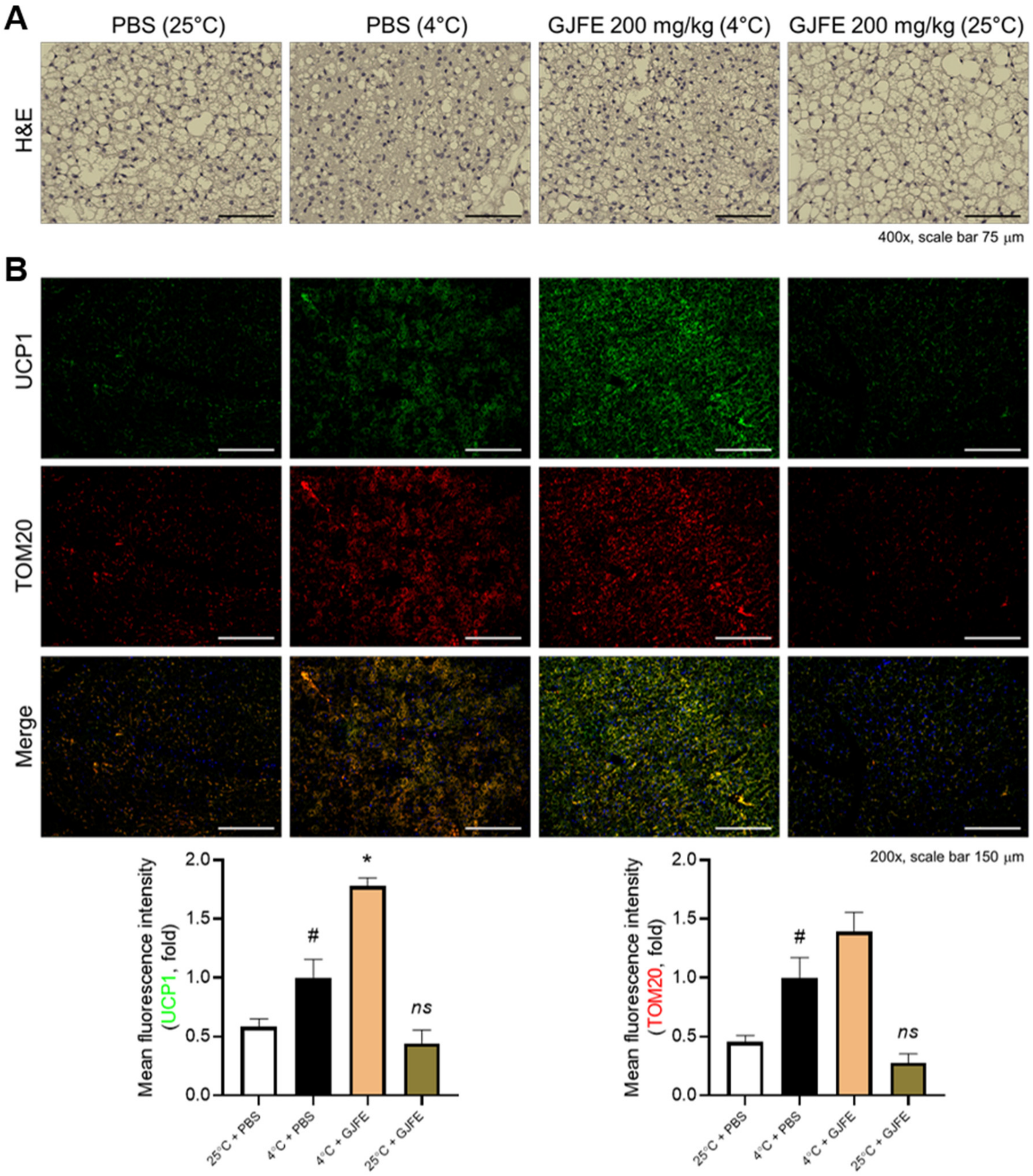

Figure S3. Effect of GJFE on BAT in cold-exposed mice. (**A**) Paraffin-embedded iBAT were stained with H&E (magnification 400×, scale bar 75 μm). (**B**) UCP1 (green) and TOM20 (red), and nuclei (blue) were detected in the BAT of the mice by immunofluorescence staining (magnification 200×, scale bar 150 μm). Intensity of the proteins was quantified using the ImageJ software. All data are expressed as the mean ± S.E.M. of the data from three or more separate experiments. $^{\#}p < 0.05$ vs. BAT of PBS-fed mice, $^{*}p < 0.05$ vs. BAT of PBS-fed and cold-exposed mice. *ns*, no significant difference. GJFE, *Gardenia jasminoides* fruit extract.

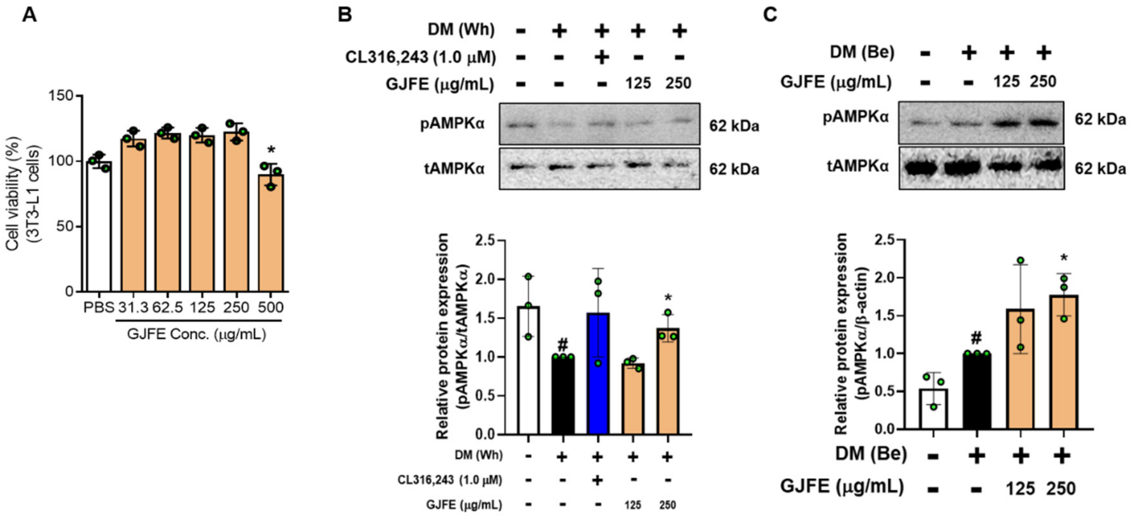

Figure S4. Effect of GJFE on cytotoxicity and protein levels of AMPK in 3T3-L1 adipocytes. (**A**) Cytotoxicity of GJFE was measured by WST-1 analysis. Phosphorylation of AMPK in (**B**) white-induced 3T3-L1 cells and (**C**) beige-induced 3T3-L1 cells was analyzed by Western blot analysis, normalized by levels of total AMPK, and quantified using the ImageJ software. All data are expressed as the mean ± S.E.M. of the data from three or more separate experiments. $^{\#}p < 0.05$ vs. DM-untreated 3T3-L1 cells, $^{*}p < 0.05$ vs. PBS-treated 3T3-L1 cell (A) or DM-treated 3T3-L1 cells (B and C). GJFE, *Gardenia jasminoides* fruit extract.

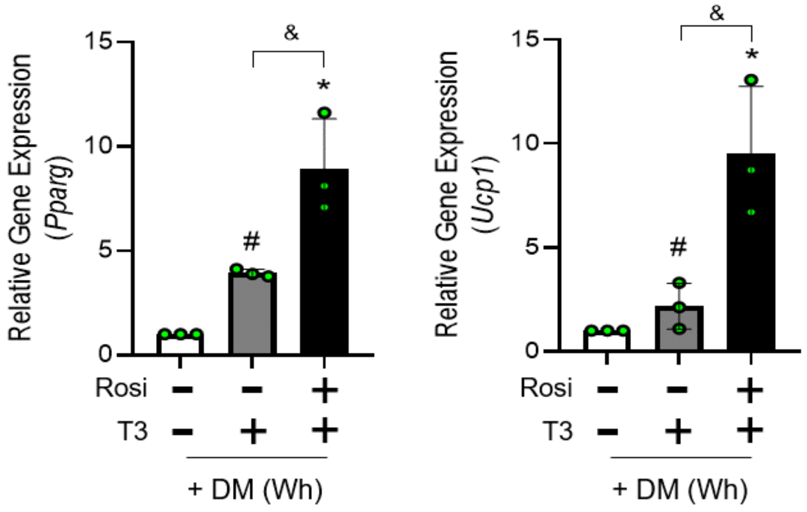

Figure S5. Effect of PPARγ agonist on mRNA expression of UCP1 in beige adipocytes. mRNA expression of *Pparg* and *Ucp1* was measured by RT-PCR analysis and normalized by *Gapdh*. All data are expressed as the mean ± S.E.M. of the data from three or more separate experiments. $^{\#}p < 0.05$ vs. DM (Wh)-treated 3T3-L1 cells, $^{*}p < 0.05$ vs. DM (Wh) and T3-treated 3T3-L1 cells. GJFE, *Gardenia jasminoides* fruit extract. Rosi; rosiglitazone.
